# Detailed statistical analysis plan for the difficult airway management (DIFFICAIR) trial

**DOI:** 10.1186/1745-6215-15-173

**Published:** 2014-05-15

**Authors:** Anders Kehlet Nørskov, Lars Hyldborg Lundstrøm, Charlotte Vallentin Rosenstock, Jørn Wetterslev

**Affiliations:** 1 Department of Anaesthesiology, Nordsjællands Hospital, Copenhagen University Hospital, Hillerød, Capital region of Denmark 3400, Denmark; 2 Copenhagen Trial Unit, Center for Clinical Intervention Research, Copenhagen University Hospital, Rigshospitalet, Copenhagen 2100, Denmark

**Keywords:** Statistical analysis plan, Cluster randomized trial, Airway management, Cluster analysis, Difficult intubation

## Abstract

**Background:**

Preoperative airway assessment in Denmark is based on a non-specific clinical assessment left to the discretion of the responsible anesthesiologist. The DIFFICAIR trial compares the effect of using a systematic and consistent airway assessment versus a non-specific clinical assessment on the frequency of unanticipated difficult airway management.

To prevent outcome bias and selective reporting, we hereby present a detailed statistical analysis plan as an amendment (update) to the previously published protocol for the DIFFICAIR trial.

**Method/Design:**

The DIFFICAIR trial is a stratified, parallel group, cluster (cluster = department) randomized multicenter trial involving 28 departments of anesthesia in Denmark randomized to airway assessment either by the Simplified Airway Risk Index (SARI) or by a usual non-specific assessment. Data from patients’ preoperative airway assessment are registered in the Danish Anesthesia Database. An objective score for intubation grading the severity, that is the severity of the intubations, as well as the frequency of unanticipated difficult intubation, is measured for each group.

Primary outcome measures are the fraction of unanticipated difficult and easy intubations.

The database is programmed so that the registration of the SARI is mandatory for the intervention group but invisible to controls.

Data recruitment was commenced in October 2012 and ended *in ultimo* December 2013.

**Conclusion:**

We intend to increase the transparency of the data analyses regarding the DIFFICAIR trial by an *a priori* publication of a statistical analysis plan.

**Trial registration:**

ClinicalTrials.gov: NCT01718561.

## Introduction

The difficult airway management trial (DIFFICAIR) is a stratified, parallel group, cluster (cluster = department) randomized and multicenter trial involving 28 departments of anesthesia in Denmark. The DIFFICAIR trial compares the effect of two regimens of preoperative airway assessment on the frequency of unanticipated difficult airway management.

Prediction of difficult airway management remains a pivotal challenge in anesthesia. Difficult tracheal intubation and difficult mask ventilation may cause serious patient complications [1-6]. By allocating experienced personnel and relevant equipment, better prediction of difficult airway management may reduce complications and, thereby, associated morbidity and mortality. There is no single predictor that is sufficiently valid in predicting difficult tracheal intubation [7-12]. However, several studies show that by combining multiple predictors of difficult tracheal intubation, the positive and the negative predictive value of the assessment increases [12]. In Denmark as well as internationally, there is no clear recommendation on how to perform airway assessment. Consequently, airway assessment in Denmark is based exclusively on the individual anesthesiologist’s preoperative clinical assessment. However, it is poorly documented how accurately this clinical assessment predicts actual airway management conditions.

The ‘Simplified Airway Risk Index’ (SARI) [13] is based on a multivariable model for airway assessment described by El-Ganzouri and colleagues enabling an estimation of the likelihood of a difficult direct laryngoscopy. The SARI contains seven individual predictors for a difficult direct laryngoscopy, each given a weighted score of 0 to 1 or 0 to 2. A summarized value of the SARI score > 3 indicates that a future direct laryngoscopy will be difficult. It is unknown, whether the SARI score predicts difficult intubation better or worse than a clinical assessment. The rationale for this trial was to prospectively compare the effect of the SARI with an non-specified clinical airway assessment on the frequency of unanticipated difficult airway management.

The target population was adult patients undergoing anesthesia. Twenty-eight departments of anesthesia were randomized to one of two groups. Intervention departments used the SARI score for preoperative airway assessment. The intervention group additionally did an assessment of risk factors for difficult mask ventilation as described by Kheterpal and colleagues [14-16]. Departments in the control group continued normal practice of preoperative airway assessment. All data were registered in the Danish Anesthesia Database (DAD). A more detailed trial protocol describing background, design and rationale has been published in *Trials*[17].

In order to prevent outcome reporting bias and results based on data-driven analysis, it is encouraged to prospectively publish a trial protocol [18,19]. The same argument applies for a prospective publication of a statistical analysis plan. Concordantly, the International Conference on Harmonization (ICH) of Good Clinical Practice (GCP) recommends that clinical trials are analyzed according to a pre-specified plan [19].

### Objective

The primary aim of the DIFFICAIR trial is to compare the effect of using a systematic airway assessment with a standard clinical airway assessment on the frequency of unanticipated difficult airway management. The null hypothesis is:

•There is no difference in the proportion of unanticipated difficult intubations when the preoperative airway assessment is based on the SARI score compared with a preoperative airway assessment based on the individual anesthesiologist’s assessment.

The alternative hypothesis is:

•The use of a systematic SARI airway assessment, registration of the SARI and risk factors for difficult mask ventilation, and continuous education in airway assessment will reduce the relative risk of a difficult intubation with 30%, corresponding to a number needed to treat (NNT) of 180 patients.

## Methods

This analysis plan has been written while the data collection from the DIFFICAIR trial was on-going and trial data non-accessible. The data analysis of the main publication will follow this plan. The statistical analysis was approved by the DIFFICAIR steering committee on 29 December 2013. The last day of data collection was 31 December 2013. The involved departments were given one additional month to ensure registration of all patients in the Danish Anesthesia Database. On 31 January 2014, the database was locked and data extracted. The statistical analysis plan was published on (www.clinicaltrials.gov) before the last data entry and before data was extracted and data management commenced.

The DIFFICAIR trial protocol has been written according to the Standard Protocol Items: Recommendations for Interventional Trials (SPIRIT) guidelines and has been public on (http://www.difficair.com) since the beginning of the trial and is registered at (www.clinicaltrials.gov) (NCT01718561). The Danish Anesthesia Database and the Danish Society of Anaesthesiology and Intensive care Medicine (DASAIM) endorsed the trial.

The trial is carried out in accordance with the Helsinki declaration. The Scientific Ethics Committee of Copenhagen County has declared that it is regarded as a quality assurance project and thus should not be reported to the committee system (Journal number: H-3-2012-FSP2). Further, the need for individual patient consent was waived. The trial is approved by The Danish Data Protection Agency (Journal number: 2007-58-0015/HIH-2011-10, I-Suite number: 02079). The reporting of the trial will be in accordance with the CONSORT 2010 statement: extension to cluster randomised trials [20].

### Randomization and sample size

Our sample size calculation was based on an adjustment for the stratification and the cluster randomized design [21,22]. Since there are no previous records of the trial’s primary outcome measure, ‘unanticipated difficult intubation’ a baseline study was conducted based on data from the DAD. In order to reject or detect a 30% relative risk reduction in the proportions of unanticipated difficult intubation between the intervention group and the control group approximately 30 departments were required in a 15 months period. Calculations were based on a maximum risk of type 1 error of 5% and risk of type 2 error of maximum 20% (80% power).

A total of 28 departments were included and randomized 1:1 using a computer generated list. The sample size calculation was based on an average cluster size of 1,611 patients. We estimated the average cluster size in the DIFFICAIR trial to approximately 2,500 patients, giving a total of 70,000 included patients during the trial period. The enhanced sample size allows for a potentially slight loss of clusters according to the power calculation, from 30 to potentially 26. Our sample size estimation may be of a conservative nature, calling for more clusters than necessary [23].

### Populations

The DIFFICAIR trial focuses on two essential elements of airway management which are tracheal intubation by direct laryngoscopy and mask ventilation. This statistical analysis plan will address analysis of the data regarding tracheal intubation. Data analysis regarding prediction of difficult mask ventilation will be handled in an analogous way, but will not be further elaborated in the present paper.

The part of the DIFFICAIR trial regarding prediction of difficult intubation comprises two populations; 1) patients that were primarily attempted intubated by direct laryngoscopy; 2) patients that were primarily attempted intubated by direct laryngoscopy (population 1) plus patients anticipated to be difficult to intubate and therefore scheduled for and intubated with an advanced method (for example, video laryngoscopic or fiber optic intubation).

The results of population 1 and 2 will be presented in one publication. Due to the extent of data, further publications presenting data from the DIFFICAIR trial will follow, but further elaboration on data analysis exceeds the content frame of this paper.

### Adjusting and stratification variables

Each cluster (department) was randomized to a control or intervention group, making this the intervention group indicator. The trial site may account for further intervention heterogeneity and will be used for adjustment in the analysis of the intervention effect. Further, a stratification variable that grouped the departments according to whether the proportion of unanticipated difficult intubation at baseline was ≥ or < 2% will be used for adjustment according to recent evidence of increased power in the analysis of stratified trials [22].

### Assumed confounding covariates

We define age; gender; ASA classification; emergency/elective procedure; Body Mass Index (BMI); and use of neuromuscular blocking agents as covariates that are possible confounders, necessitating adjusted analyses of the primary outcome and pre-defined subgroup analyses.

### Primary outcomes

The primary outcome measures are:

1. The fraction of unanticipated difficult intubations = all intubations with unanticipated difficulties (False negative)/all patients primarily (attempted) intubated by direct laryngoscopy.

2. The fraction of unanticipated easy intubations = all intubations with anticipated difficulties that were easy (False Positive)/all patients primarily (attempted) intubated by direct laryngoscopy.

The two primary outcomes are linked and simultaneous low fractions are desirable for the optimal prediction of a difficult intubation.

### Secondary outcomes

1. 48-hour mortality.

2. 30-day mortality.

3. The fraction of anticipated difficult intubations planned for, and intubated by an advanced method/all patients (attempted) intubated.

4. The fraction of unanticipated difficult intubations (False Negative)/all difficult intubations ((False negative) + (True Positive)).

5. Sensitivity of the prediction of a difficult/easy intubation.

6. Specificity of the prediction a difficult/easy intubation.

7. Predictive value of a positive prediction of difficult/easy intubation.

8. Predictive value of a negative prediction of difficult/easy intubation.

9. Positive Likelihood Ratio = (Sensitivity/(1-Specificity)).

10. Negative Likelihood Ratio = ((1-Sensitivity)/Specificity).

11. The Receiver Operating Characteristic (ROC) curve. A graphical representation of sensitivity as a function of (1-Specificity).

Outcomes 5 to 10 are measured for both intervention groups.

Outcome 11 will be measured on relevant non-binary predictors.

### Datapoints

#### Baseline covariates

Individual level:

1. Sex

2. Age

3. Height

4. Weight

5. BMI

6. American Society of Anesthesiologists (ASA) Classification

7. Use of neuromuscular blocking agents

8. Hospital unit

9. Region

10. Anticipated difficult tracheal intubation

11. Anticipated difficult mask ventilation

12. Scheduled airway

13. Priority: emergency/elective

14. Surgical procedure codes

15. Intubation score

16. Mask ventilation score.

#### Intervention covariates

1. Mouth opening

2. Thyro-mental distance

3. Modified Mallampati classification

4. Jaw protrusion

5. Neck mobility

6. Previous difficult airway management

7. Number of completed risk factors

8. The calculated SARI score

9. Dichotomized SARI score (<or ≥ 4)

10. Snoring

11. Sleep apnoea

12. Presence of beard

13. Changes in the neck due to radiation.

#### Cluster level summaries

1. Mean cluster size

2. Mean number of intubated patients

3. Fraction of private hospitals

4. Mean fraction of unanticipated difficult intubation

5. Mean fraction of unanticipated easy intubation

6. Age

7. BMI

8. ASA classification.

### Definition of difficult intubation

In the DAD, an intubation score is programmed based on numbers of intubation attempts and use of equipment.

1. A maximum of two intubation attempts - only by direct laryngoscopy.

2. A maximum of two intubation attempts in which other intubation equipment or assistive devices for direct laryngoscopy is used (for example, video laryngoscope).

3. Three intubation attempts or more - regardless of intubation method.

4. Intubation failed despite attempting.

Tracheal intubation by direct laryngoscopy is pre-defined in the DAD as easy by a score = 1 and difficult by a score ≥ 2. In our primary analyses and sample size calculation we employ the same definition.

### General analysis principles

1. Unless otherwise stated, all main analyses will compare the two intervention groups using intention-to-treat (ITT) [24].

2. In order to ensure a correct type 1 error risk, all main analyses will account for the clustered design of the trial and the stratification variable [25-27]. Analyses will be based on individual patient level data but clustering of patients and the stratification variable will be accounted for in a generalized estimating equation.

3. In all analyses, a maximum level of 5% (two-sided) type 1 error will be regarded as statistically significant unless otherwise stated.

4. Main analyses will be according to ITT adjusted for cluster and stratification variables. Sensitivity analyses will be performed adjusted and unadjusted for the prior listed potential confounding covariates. We will discuss if results differ from the main analyses. The conclusion of the trial will be based on the primary analyses.

5. Test of interaction will be applied for subgroup analyses.

6. Risks are reported as relative risks and odds ratios. When relative risks are calculated from odds ratios with 95% confidence interval (CI) it will be done according to Zhang and Yu [28].

7. For missing data exceeding a rate of 5%, and with a statistical significant Little’s test, indicating that the missing data is not a completely random sample of the total data, point estimates with 95% CI will be calculated using a worst/best case scenario imputation on the missing values. If the imputation of a worst/best case scenario implies different conclusions, multiple imputations will be performed on the missing values assuming missingness at random [29]. Unadjusted and complete case analyses will also be presented.

8. In order to avoid rejecting a true null hypothesis we will address the problem of multiplicity by Bonferroni adjustments on the secondary outcome measures. If unadjusted analyses are insignificant (*P* > 0.05), Bonferroni adjustments will not be applied. In case the adjustment changes an unadjusted significant *P*-value to a non-significant *P*-value, this will be discussed.

9. To ensure complete objectivity, the author (AN) will be blinded for the intervention group in the primary outcome analysis and, as far as this is possible, for analyses of secondary outcomes. However, analyses of the predictive properties of the SARI will require un-blinding of AN. After data collection, a third party data manager will generate a complete dataset with blinded coding of the intervention groups and other variables possibly revealing the intervention. The statistician performs the primary outcome analysis on this data set. If the primary outcome differs between groups, we will construct different conclusions reflecting the results, considering that significant differences of the intervention could both be of benefit or harm. After writing the conclusions, we will uncover the code of the blinding, and subsequently the correct conclusion will be employed [30].

### Statistical analyses

#### Trial profile

The flow of study participants will be displayed in a Consolidated Standards of Reporting Trials (CONSORT) diagram at a cluster level and at individual level. The number of clusters fulfilling the inclusion criteria, and the number of clusters included in primary and secondary analyses, will be presented. The number of patients who fulfilled study inclusion criteria as well as the number included in the primary and secondary analyses will be reported. Reasons for exclusions of clusters and patients in the primary and secondary analyses will be reported.

#### Primary outcome

Frequencies and percentages per group will be reported with a 95% CI. The primary outcome is presented as odds ratios and relative risk ratios.

The primary analysis of the primary outcome will be adjusted for the stratification- and the cluster-variable performed according to the ITT principle including patients that met the inclusion- and not the exclusion-criteria. A generalized estimating equation will be used. Intervention group and stratification variable are regarded as fixed effects and trial site is regarded as random effects in the model. We will test the robustness of the results by repeating the analyses with a mixed effects model and finally with a standard *t*-test comparing the means of the outcome at department level in each intervention group.

The first sensitivity analysis of the primary outcome will be adjusted for the stratification- and cluster-variables as well as baseline covariates assumed as confounders incorporated in a generalized estimating equation.

In the second sensitivity analysis of the primary outcome, we will employ a different cut-off value for difficult intubation using ≥ 3 instead of ≥ 2 as the definition of difficult intubation.

Further sensitivity analyses of the primary outcome will compare the patients in the control group that met the inclusion- and not the exclusion-criteria with patients in the intervention group who received the protocoled intervention. That is, a *per protocol* analysis of control group versus the subgroup in the intervention group that had a sufficiently registered SARI. Interaction test will be performed in the intervention group between patients receiving sufficient/insufficient SARI registration.

#### Secondary outcomes

Frequencies, proportions, percentages, odds and risk ratios are presented with a 95% CI for each group. A chi-squared test is used to assess the effect of the intervention on binary outcomes. For categorical outcomes and the adjusted analyses, logistic regression analysis or generalized estimating equations will be performed.

### Baseline comparisons of patient characteristics

Baseline characteristics are presented for each intervention group. Frequencies, proportions and percentages will be used to summarize discrete variables. In case of missing values, percentages are presented with the actual denominator and otherwise calculated according to the number of participating patients. Continuous variables are summarized using standard measures of central tendency and dispersion using either mean ± SD for data with normal distribution or median and interquartile range for non-normally distributed data.

### Baseline comparisons of cluster characteristics

Cluster characteristics are presented for each group, control and intervention. Unless otherwise stated, data will be presented as means with SD for data with normal distribution or median and interquartile range for non-normally distributed data.

### Outline of figures and tables

The first figure will be a CONSORT flow chart on individual patient level and cluster level. A second figure will illustrate the SARI score and tutorial instruments. A third figure will demonstrate the registration in the DAD, including the intubation score. A fourth figure will present baseline data from each intervention group on individual and cluster level and a fifth figure will be outlining the main outcome results for each intervention group.

## Discussion

In order to avoid outcome reporting bias and data-driven results this paper presents the detailed statistical analysis plan for the main publication of the DIFFICAIR trial. The DIFFICAIR trial raises two important questions, which are: is it possible via the intervention to reduce the frequencies of difficult intubation and/or difficult mask ventilation? This plan only addresses the statistical analyses of the population of intubated patients because our sample size calculations were based on this population. Secondly, the SARI was developed as a prediction tool for difficult intubation. Finally, the extent of data necessitates several publications.

By adjusting our primary outcome analysis for different design variables, such as clustering and stratification, we strive to eliminate inflated type 1 error rates as a consequence of the trial design. A generalized estimating equation is applied based on an evaluation of each variable as having random or fixed effects [31,32].

When multiple comparisons are performed between two groups, you may risk accepting an intervention effect erroneously (type 1 error). There are several approaches that deal with multiple testing. We will employ Bonferroni adjustments on the secondary outcome measures in order to evaluate, identify and discuss dubious significant outcomes that may be due to statistical multiplicity.

The value of a diagnostic test is usually presented as sensitivity and specificity. We have chosen (1 - total accuracy), that is the proportion of unanticipated difficult intubations (False Negative, FN) and the proportion of unanticipated easy intubations (False Positive, FP). Both scenarios are of clinical relevance since the FNs are at risk of hypoxia, increased morbidity and even death, while the FPs are at risk of being imposed unnecessary discomfort by, for example, awake intubation. At the same time, both the FNs and FPs can take up unnecessary resources. Sensitivity and specificity are more difficult to interpret intuitively. Consequently, we chose to present more transparent primary outcomes. Using proportions of unanticipated difficult intubation allowed us to perform a baseline cohort study, on which we based our sample size and power calculations.

By publishing this paper, where we pre-specify our methods and analyses, it is our hope that the results from the DIFFICAIR trial will be as transparent and robust as possible.

## Conclusion

This paper presents the principles of analyses of the main outcomes in the DIFFICAIR trial for the first publication based on patients who underwent intubation. Our approach aims to minimize the risk of data-driven results and outcome reporting bias.

## Abbreviations

ASA: American Society of Anesthesiologists; BMI: Body Mass Index; CI: confidence interval; DAD: Danish Anesthesia Database; FN: False Negative; FP: False Positive; GCP: Good Clinical Practice; ICH: International Conference on Harmonization; ITT: intention-to-treat; NNT: number needed to treat; ROC: Receiver Operating Characteristic; SARI: Simplified Airway Risk Index; SD: standard deviation; SPIRIT: Standard Protocol Items: Recommendations for Interventional Trials.

## Competing interests

The authors declare no financial or non-financial competing interests.

## Authors’ contribution

AN, LL, and JW proposed the statistical analysis plan. AN drafted the manuscript. AN, LL, CR, and JW participated in the design of the trial. AN conducted the coordination of the trial. AN, LL, CR, and JW read, amended and approved the statistical analysis plan and the final manuscript.
